# Cardiac Magnetic Resonance Imaging for Diagnosis of Cardiac Sarcoidosis: A Meta-Analysis

**DOI:** 10.1155/2018/7457369

**Published:** 2018-12-17

**Authors:** Jianxiong Zhang, Yunxiao Li, Qiufen Xu, Bo Xu, Haoyan Wang

**Affiliations:** Department of Respiratory Medicine, Beijing Friendship Hospital, Capital Medical University, Beijing 100050, China

## Abstract

**Background:**

Cardiac magnetic resonance imaging (CMR) is an effective technique for the diagnosis of cardiac sarcoidosis (CS). The efficacy of CMR versus the Japanese Ministry of Health and Welfare (JMHW) guidelines considered as standard criterion for the diagnosis of CS remains to be elucidated.

**Methods:**

In this systematic review and meta-analysis, we aimed at assessing the diagnostic accuracy of CMR in cardiac sarcoidosis. We searched on PubMed from January 1, 1980, to March 28, 2018, on Embase from January 1, 1980, to March 29, 2018, and on the Cochrane Library from January 1, 1980, to April 1, 2018, using a strategy based on the search terms (sarcoidosis and magnetic resonance imaging) independently. We analyzed the data obtained with Revman 5.3 and Stata 14.0 software.

**Results:**

Eight studies with a total of 649 participants met the inclusion criteria, and data were extracted. CMR had an overall sensitivity of 0.93 (95% confidence interval (CI), 0.87–0.97) and specificity of 0.85 (95% CI, 0.68–0.94) for the diagnosis of cardiac sarcoidosis. The area under the summary receiver operating characteristic (SROC) curve was 0.95 (95% CI, 0.93–0.97). The subgroup analysis via public year showed that studies between 2011 and 2017 had an overall sensitivity of 0.95 (95% CI, 0.88–0.98) and specificity of 0.92 (95% CI, 0.49–0.99), with an area under the SROC curve being 0.96.

**Conclusions:**

The results of this meta-analysis suggest that CMR could be used for the diagnosis of cardiac sarcoidosis and screening of patients suspected of CS. With the improvement of the technique, the diagnostic accuracy of MRI has improved.

## 1. Introduction

Sarcoidosis is a multisystem inflammatory disease characterized by inflammation, characteristic noncaseating granuloma formation, and organ dysfunction [1]. The lungs and pulmonary lymph nodes are the most commonly affected, but other tissues including the skin, eyes, central nervous system, liver, spleen, skeleton, and heart can also be involved. It was once believed that there was a low incidence of sarcoidosis with cardiac involvement. However, recently, high rates of cardiac involvement and adverse outcomes have been reported among Japanese patients, with 5-year mortality rates of CS ranging between 25 and 67% [2–6]. CS can be life-threatening and cause conduction defects, fatal arrhythmias, congestive heart failure, and sudden cardiac death, which is determined by the degree of localization and the severity of the disease [2, 7, 8]. It has been reported that an early initiation of corticosteroid therapy can minimize adverse outcomes, so it is crucial to screen patients with suspected cardiac involvement [9–11]. However, cardiac sarcoidosis may have no clinical manifestations or nonspecific presentation, so diagnosis based on clinical criteria may be difficult. Advanced imaging modalities, including cardiac magnetic resonance (CMR) and positron emission tomography (PET), may help in both the diagnosis and assessment of response to treatment for cardiac sarcoidosis. This systematic review and meta-analysis were performed to be helpful for the diagnosis of CS.

## 2. Methods

### 2.1. Data Collection

We did not restrict the searches to particular study designs, or publication dates. We searched on PubMed from January 1, 1980, to March 28, 2018, on Embase from January 1, 1980, to March 29, 2018, and on the Cochrane Library from January 1, 1980, to April 1, 2018, using a strategy based on the search terms (sarcoidosis and magnetic resonance imaging) independently, detailed in Appendix 1, and included all studies of the accuracy of diagnostic tests. Studies were excluded if they did not contain sufficient information to complete a 2 × 2 table. For studies performed on the same population, we included the most recent results. The results of the literature search, literature screening, record eligibility, and study quality were assessed independently by 2 reviewers (J. Z. and Y. L.). Disagreements were resolved by adjudication by the other authors. Extracted data were used to create forest plots of sensitivity and specificity, with 95% confidence intervals (CIs), using Review Manager 5.3 (Nordic Cochrane Center, Copenhagen, Denmark). Given the data and diagnostic-threshold variability, a random-effects hierarchical summary receiver operating characteristic (SROC) model, fit the results onto an SROC curve using Stata 14.0 statistical software (Stata Corporation, College Station, TX, US).

### 2.2. Study Selection

The initial database created from the literature search was screened by two reviewers blindly. If the two reviewers had different opinions, disagreements were resolved by means of a discussion between them. Studies were included if they met the following criteria: (1) evaluation of the diagnostic accuracy of CMR for sarcoidosis; (2) use of a sample size of ≥20. Studies were excluded if they did not use CMR to evaluate cardiac sarcoidosis, or if they contained insufficient data.

### 2.3. Data Extraction and Quality Assessment

Two reviewers collected data from the primary studies independently. For each test, the data were classified as positive or negative for the cardiac magnetic resonance imaging detection of cardiac sarcoidosis and sensitivity and specificity estimates were calculated. Data were recorded on a standard data extraction form. Two authors independently assessed the quality of each included study using the risk of bias tool in Review Manager 5.3 software.

### 2.4. Statistical Analysis

We plotted study estimates of sensitivity and specificity on forest plots and in receiver operating characteristic (ROC) space. Because our focus of inference was summary points, we used a bivariate model to jointly summarize sensitivity and specificity, through the inclusion of random effects for the logit sensitivity and specificity parameters of the bivariate model [12, 13]. We also assessed the influence of statistical heterogeneity on the pooled estimates of the individual results using the *I*^2^ test. If *I*^2^ value was ≥50%, it indicated significant heterogeneity. A value of *P* < 0.05 was considered significant for the chi-squared test of heterogeneity. We performed sensitivity and specificity analysis in which a subgroup analyses of the publication date were performed. The presence of publication bias was evaluated by the Deeks funnel plot asymmetry test using Stata 14.0. We calculated the posttest probabilities using the pretest probability, and the summary positive and negative likelihood ratios, evaluated using the Fagan plot analysis command in Stata 14.0.

## 3. Results

### 3.1. Study Characteristics

**Figure 1:**
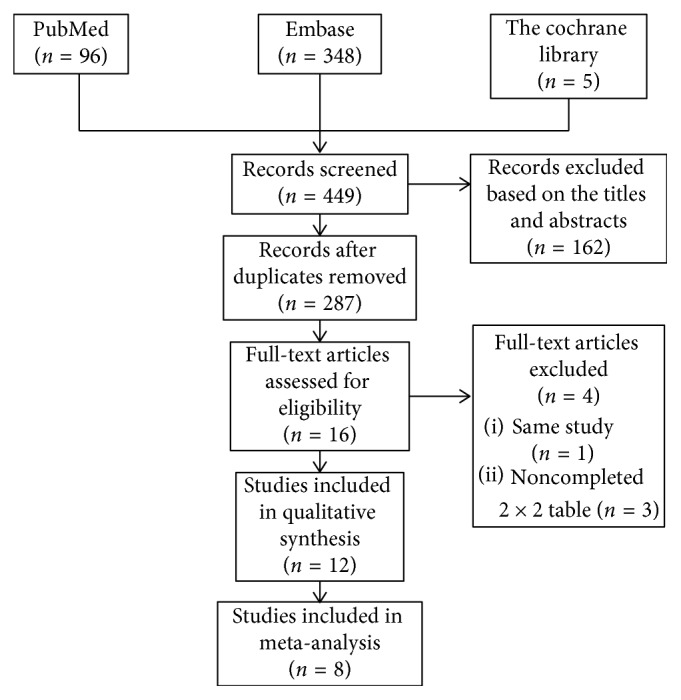
The flow of the process of identifying eligible studies.

We collected 449 records from PubMed, Embase, and the Cochrane Library. Titles were screened to exclude 162 duplicates from the initial 449 records. Screening of the titles, abstracts, study types, and full texts of the remaining 287 records resulted in the identification of 12 qualifying studies. Finally, we obtained 8 studies for the systematic review and meta-analysis [14–21]. The flow of the process of identifying eligible studies is presented in Figure 1. We compared CMR to the Japanese Ministry of Health and Welfare (JMHW) guidelines, which are accepted as the reference standard for this condition, and it was used here in 8 of the included studies. We excluded four studies because their reference standards were endomyocardial biopsy (EMB) and PET/CT.

**Figure 2:**
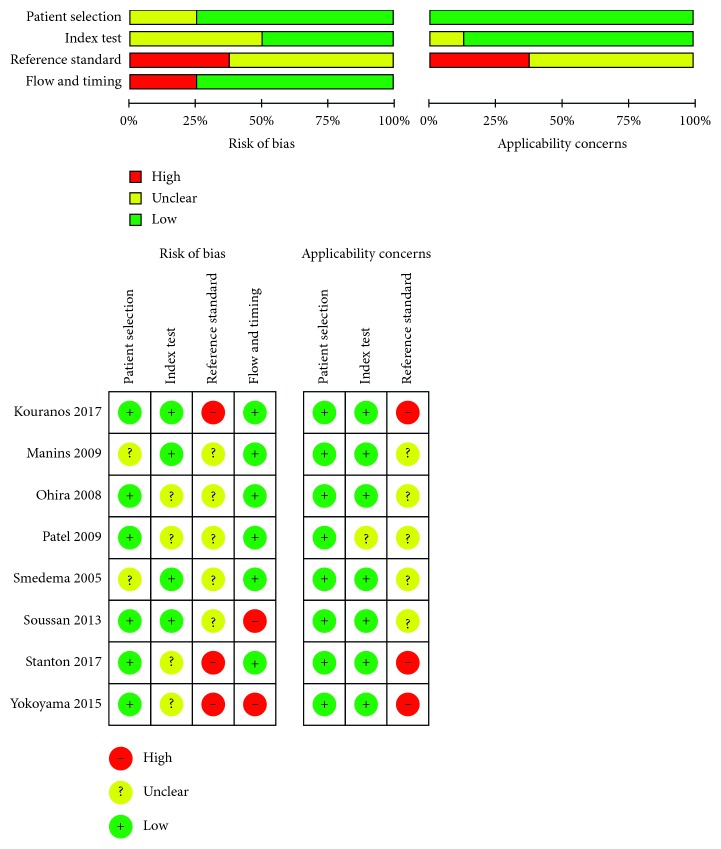
Risk of bias of the 8 included studies.

The main information and characteristics from the included studies are summarized in Table 1. Of these studies, 3 were conducted in Europe, 2 in Australia, 2 in Asia, and 1 in North America. Of these, 4 studies were published between 2005 and 2009, and the remaining 4 were published between 2013 and 2017. There were no randomized controlled trials and all the studies were observational, mainly of cross-sectional design. Study quality of the included studies was presented in Figure 2.

**Figure 3:**
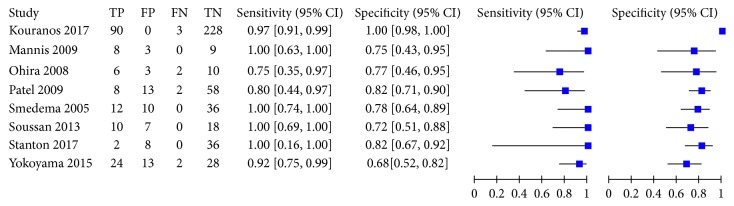
Forest plots of sensitivity and specificity. CMR had an overall sensitivity of 0.93 (95% CI, 0.87–0.97) and specificity of 0.85 (95% CI, 0.68–0.94) in the diagnosis of cardiac sarcoidosis.

**Figure 4:**
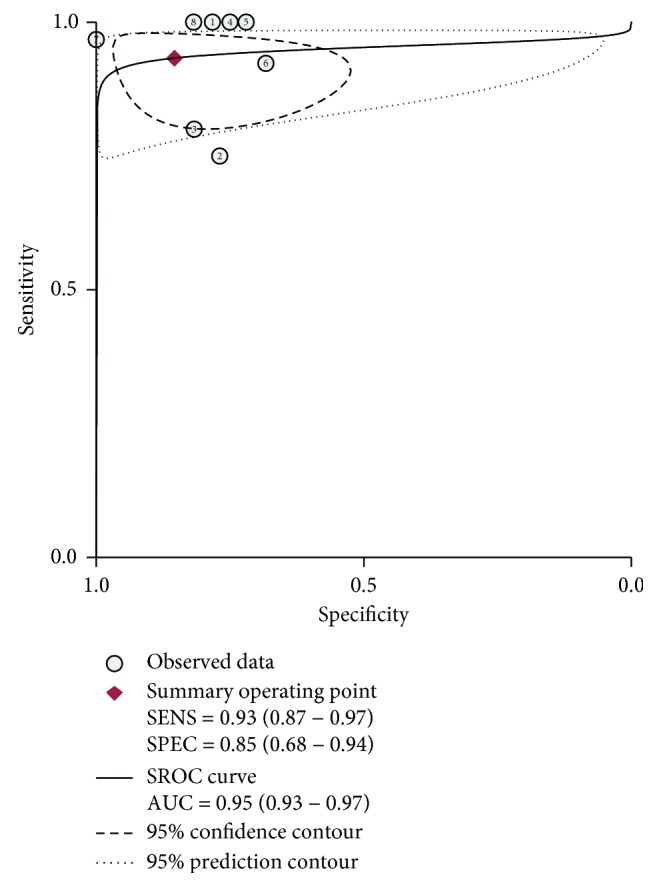
SROC curve. A random-effect SROC model was used, given the data and diagnostic-threshold variability to fit a single symmetric SROC curve. The area under the SROC curve was 0.95 (95% CI, 0.93–0.97). The overall diagnostic odds ratio was 81 (95% CI, 20–332).

**Figure 5:**
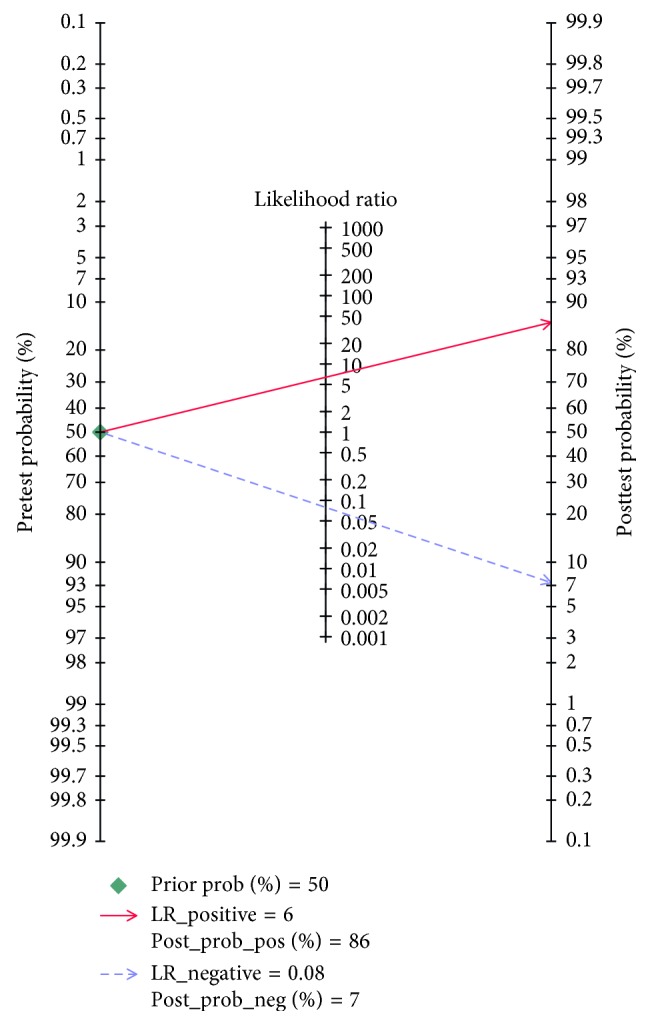
The Fagan plot analysis showed the pretest probability is 50, the positive likelihood is 6, the probability of posttest is 86, the negative likelihood ratio is 0.08, and the probability of the posttest is 7.

This meta-analysis encompassed eight studies with a total of 649 participants; CMR had an overall sensitivity of 0.93 (95% CI, 0.87–0.97) and specificity of 0.85 (95% CI, 0.68–0.94) in the diagnosis of cardiac sarcoidosis (Figure 3). The *I*^2^ statistic was 36.70 (95% CI, 0.00–88.40) and 87.59 (95% CI, 80.36–94.83), suggesting that there was no significant heterogeneity for sensitivity but there was significant heterogeneity for specificity. A random-effect SROC model was used given the data and diagnostic-threshold variability to fit a single symmetric SROC curve (Figure 4). The area under the SROC curve was 0.95 (95% CI, 0.93–0.97). The overall diagnostic odds ratio was 81 (95% CI, 20–332). The Fagan plot analysis (Figure 5) showed the following: the pretest probability, 50; positive likelihood, 6; probability of posttest, 86; negative likelihood ratio, 0.08; and the probability of the posttest, 7.

**Figure 6:**
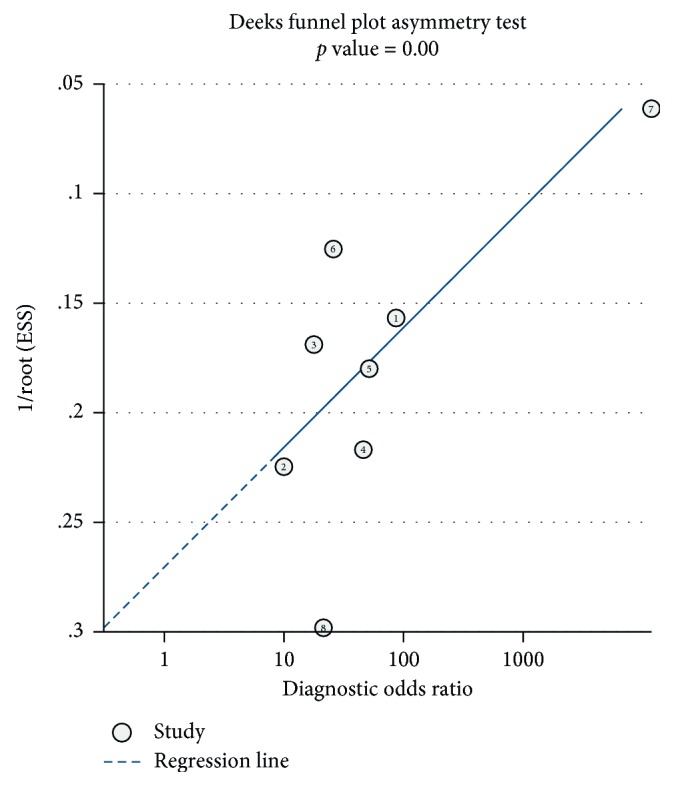
The Deeks funnel plot asymmetry test of publication bias. The Deeks funnel plot asymmetry test of publication bias of the diagnostic odds ratios revealed publication bias existed (*p* < 0.00).

**Figure 7:**
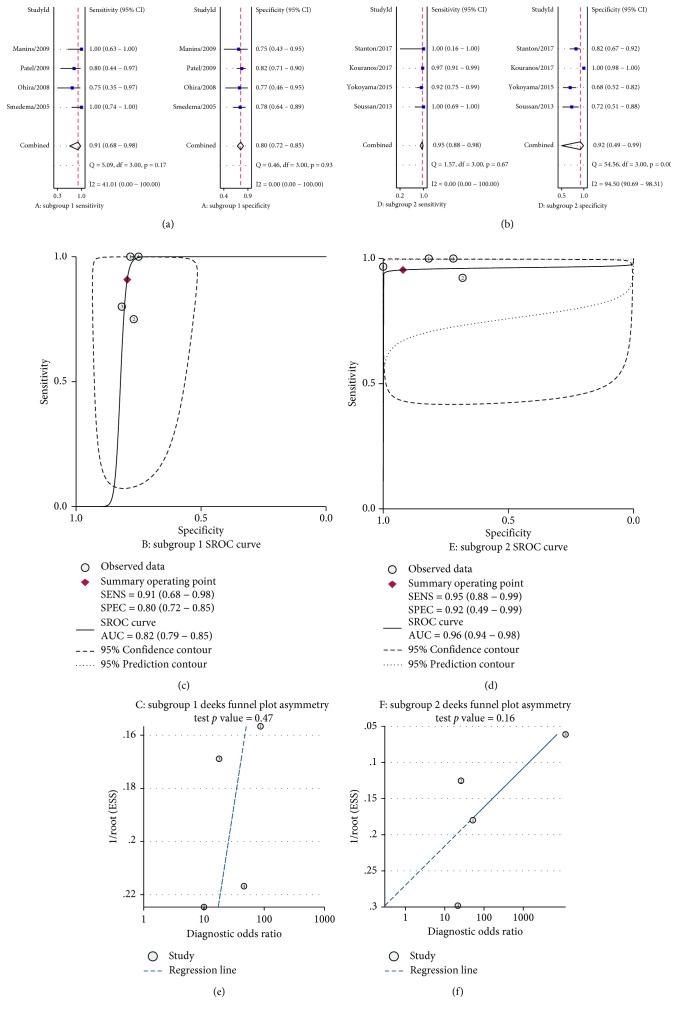
Forest plots of sensitivity and specificity, SROC curves, and the funnel plot asymmetry test based on the subgroup.

The Deeks funnel plot asymmetry test of publication bias of the diagnostic odds ratios revealed publication bias existed (*P* < 0.00, Figure 6). So we performed subgroup analyses via public year, one group (subgroup 1) public year between 2005 and 2011, and another group (subgroup 2) published year between 2011 and 2017. Subgroup analysis showed that subgroup 1 had an overall sensitivity of 0.91 (95% CI, 0.68–0.98) and specificity of 0.80 (95% CI, 0.72–0.85) in the diagnosis of cardiac sarcoidosis (Figure 7(a)). The *I*^2^ statistic was 41.01 (95% CI, 0.00–100.0) and 0.00 (95% CI, 0.00–100.00), suggesting less heterogeneity in specificity. A random-effect SROC model was used, given the data and diagnostic-threshold variability to fit a single symmetric SROC curve (Figure 7(b)). The area under the SROC curve was 0.82 (95% CI, 0.79–0.85). The Deeks funnel plot asymmetry test of publication bias of the diagnostic odds ratios revealed publication bias was decreased (*P*=0.47) (Figure 7(c)). And for the subgroup 2, an overall sensitivity of 0.95 (95% CI, 0.88–0.98) and specificity of 0.92 (95% CI, 0.49–0.99) were observed in the diagnosis of cardiac sarcoidosis (Figure 7(d)). The *I*^2^ statistic was 0.00 (95% CI, 0.00–100.0) and 94.50 (95% CI, 90.69–98.31). A random-effect SROC model was used, given the data and diagnostic-threshold variability to fit a single symmetric SROC curve (Figure 7(e)). The area under the SROC curve was 0.96 (95% CI, 0.94–0.98). The Deeks funnel plot asymmetry test of publication bias of the diagnostic odds ratios indicated that publication bias has decreased (*P*=0.16) (Figure 7(f)).

## 4. Discussion

The diagnosis of CS lacks reliable and specific tools, particularly during the early stages of the disease [24]. Cardiac magnetic resonance can facilitate both the diagnosis and assessment of response to treatment for cardiac sarcoidosis. CMR holds promise for the detection of regional interstitial edema and scarring consistent with CS. Our meta-analysis demonstrated that the diagnostic accuracy of MRI is improved with the improvement of the technique. The Fagan plot analysis showed the probability of cardiac sarcoidosis to be 86% when a patient had positive CMR results and 7% when negative. The diagnostic accuracy of CMR has increased notably in the recent years. Mapara et al. reported a meta-analysis that compared MRI with JMHW and included eight articles published before 2011; the pooled sensitivity was found to be 84% and specificity, 85%. In our meta-analyses, we included four studies before 2011; the pooled sensitivity was found to be 91% and specificity, 80%. However, the differences may be as a result of the search strategy and selection criterion.

The guidelines of JMHW are the worldwide standard used to diagnose CS [25]. Most of the studies included here used JMHW standards as the criteria for diagnosis. However, JMHW criteria do not give heavy weighting to gadolinium-enhanced CMR. Recently, the Heart Rhythm Society (HRS) listed specific late gadolinium enhancement (LGE) patterns on CMR and cardiac uptake on PET as major criteria for diagnosis of CS [26]. In addition, myocardial biopsy is considered the gold standard because it is highly specific when positive, but is invasive and has poor sensitivity for cardiac involvement, with lesions usually showing a patchy distribution [27, 28]. EMB with voltage guidance may increase the accuracy [29]. Sobol et al. and Yoshida et al. evaluated the accuracy of the test by comparing CMR with EMB; CMR showed high sensitivity and a negative predictive value for the assessment of EMB-evidenced myocardial pathology [30, 31].

Clinical assessment of cardiac sarcoidosis includes history, physical examination, electrocardiography, 24-h Holter monitoring, and echocardiography [32, 33]. Electrocardiography manifestations of CS are clinically significant. Patients are likely to experience adverse outcomes if they also suffer from atrioventricular blockage, ventricular tachycardia, and ventricular fibrillation [26, 34]. The frequent type of blockage is third-degree atrioventricular blockage [27]. Echocardiography may present the ventricular wall motion abnormalities, which indicate that the disease is at an advanced stage [35].

The typical pathology in CS is patchy edema and granulomatous infiltration of the myocardium. The inflammatory phase is characterized by focal wall thickening for infiltration or edema, combined with wall motion abnormalities, and focal myocardial thickening which can be seen as increased signal intensity on T2-weighted images and early gadolinium enhancement [36–38]. Recent reports suggest that high T2 values represent early stages of the disease, which may be reversible with treatment [37, 39]. It mainly shows thinning of the wall and delayed gadolinium enhancement, representing myocardial damage, scarring, necrosis, and fibrosis in the chronic phase [6, 40, 41]. For the myocardium, the most frequently involved area is the ventricular septum, followed by the inferior wall, anterior left ventricle, right ventricle, lateral left ventricle, and the papillary muscles [22, 42]. When the septum is involved, abnormal conduction may take place [20]. In addition, gadolinium enhancement is useful to assess the response after therapy and detect the regions of irreversible injury or fibrosis [43, 44].

CMR has currently one limitation: it cannot be performed in patients with CS who carry cardiac devices, such as cardiac defibrillators (AICD) or pacemakers, which are contraindications to CMR [35]. Another limitation is the inability to use gadolinium to image patients with renal impairment. When LGE-CMR cannot be performed due to renal insufficiency or bronchial asthma, T2BBWI may be useful [39].

PET can also be useful to diagnose CS, and studies indicated that PET could be useful as a marker of disease activity and adverse events and thus help guide therapy [18, 45]. Several studies compared late enhancement in CMR and 18F-fluoro-2-deoxy-d-glucose (18F-FDG) uptake in PET to identify cardiac involvement, PET through identification of active inflammation, and MRI through identification of mature fibrosis or scar [15, 18, 23, 45]. When CMR and 18F-FDG PET were compared with the JMHW guidelines, CMR showed higher specificity but lower sensitivity and a better negative predictive value, indicating it might be superior for ruling out CS [23, 40]. Freeman et al. developed a scoring system, which uses common clinical trials to predict positive-imaging findings; using cMRI or FDG-cPET, they found that the scoring system seemed to be driven more by the findings from cMRI than FDG-cPET [21]. The advantages of 18F-FDG PET include the biological nature of the imaging signal and the feasibility of imaging patients with electrical devices who cannot undergo CMR [41]. The disadvantages of 18F-FDG PET include radiation exposure, potential pitfalls of inadequately suppressed physiologic cardiac uptake, and the inability to detect smaller regions of myocardial damage. The combination of both PET scan and MRI testing can improve diagnostic performance [46, 47].

## 5. Conclusion

The results of this meta-analysis suggest that CMR could be used in the diagnosis of cardiac sarcoidosis and screening of the patients suspected with CS. With the improvement of the technique, the diagnostic accuracy of MRI improved. When cardiac involvement is suspected, it is crucial to thoroughly screen the patients. An early initiation of corticosteroid therapy can minimize adverse outcomes.

## Figures and Tables

**Table 1:** Review of the literature.

Study	Year	Country	Study population	Standard clinical investigations	CMR diagnostic criteria	Reference test
Smedema et al. [22]	2005	The Netherlands	58 patients with histologically proven pulmonary sarcoidosis	ECGHolterUCGSPECT	Hyperenhancement on DE-MRI	JMHW
Ohira et al. [23]	2008	Japan	21 consecutive patients with suspected cardiac sarcoidosis	ECGHolterUCG sACE 99mTc-sestamibi scintigraphy 18F-FDG PET stress ECG (if needed) nuclear cardiac testing (if needed) coronary angiography (if needed)	High signal intensity on T2WI hyperenhancement on DE-MRI	JMHW
Patel et al. [18]	2009	America	81 consecutive biopsy-proven sarcoidosis patients	ECG cardiac-imaging study (at least one, non-CMR) cardiac biopsy (if performed) X-ray coronary angiography if performed	Hyperenhancement on DE-MRI	JMHW
Manins et al. [19]	2009	Australia	20 consecutive biopsy-proven sarcoidosis patients with a suspicion of CS	ECGHolterUCGGallium -67 radionuclide investigation (if performed) PET (if performed) cardiac biopsy (if performed)	Hyperenhancement on DE-MRI showed regional wall motion abnormalities with regional fibrosis and edema	JMHW
Soussan et al. [15]	2013	France	35 consecutive biopsy-proven sarcoidosis patients with a suspicion of CS	ECGHolterUCGPET /CTSPECT (if performed)	Hyperenhancement on DE-MRI spared the subendocardium and remained limited to the middle or epicardial portion of the myocardic wall or transmural	JMHW
Yokoyama et al. [17]	2015	Japan	125 consecutive patients with suspected CS	sACE C-reactive protein BNP ECG (if needed) UCG (if needed) PET myocardial perfusion scintigraphy (if needed)	Hyperenhancement on DE-MRI	JMHW
Kouranos et al. [14]	2017	Greece and the United Kingdom	321 consecutive biopsy-proven sarcoidosis patients (all Caucasians)	ECG Holter UCG BNP sACE 67Gallium scintigraphy (within 3 months) chest Radiograph (within 3 months) pulmonary Function tests (within 3 months)	Hyperenhancement on DE-MRI and regional wall motion abnormalities	HRS consensus criteria and JMHW
Stanton et al. [16]	2017	Australia	46 consecutive patients with biopsy-proven sarcoidosis	ECG UCG Holter (if performed) exercise stress tests (if performed) PET (if performed)	Hyperenhancement on DE-MRI	JMHW

ECG: electrocardiogram; UCG: ultrasound cardiogram; SPECT: perfusion single photon emission computed tomography; DE-MRI: delayed enhancement magnetic resonance imaging; JMHW: the guidelines of the Japanese Ministry of Health and Welfare; sACE: serum angiotensin-converting enzyme; 18F-FDG: 18F-fluoro-2-deoxy-d-glucose, BNP: B-type natriuretic peptide.

## Data Availability

The meta-analysis data used to support the results of this study are included in the article.

## Appendix

### Search Strategy

PubMed (2018-3-28), Embase (2018-3-29), and the Cochrane Library (2018-4-1) using the following search terms (sarcoidosis OR sarcoidoses OR “Besnier-Boeck-Schaumann Syndrome” OR “Besnier Boeck Schaumann Syndrome” OR “Syndrome, Besnier-Boeck-Schaumann” OR “Boeck Disease” OR “Schaumann's Syndrome” OR “Schaumann's Syndromes” OR “Syndrome, Schaumann's” OR “Boeck's Sarcoid” OR “Boeck Sarcoid” OR “Boecks Sarcoid” OR “Sarcoid, Boeck's” OR “Schaumann Disease” OR “Disease, Schaumann” OR “Schaumann Syndrome” OR “Syndrome, Schaumann” OR “Besnier-Boeck Disease” OR “Besnier Boeck Disease” OR “Boeck's Disease” OR “Boecks Disease”) AND (“Magnetic Resonance Imaging” OR “Imaging, Magnetic Resonance” OR “NMR Imaging” OR “Imaging, NMR” OR “Tomography, NMR” OR “Tomography, MR” OR “MR Tomography” OR “NMR Tomography” OR “Steady-State Free Precession MRI” OR “Steady State Free Precession MRI” OR “Zeugmatography” OR “Imaging, Chemical Shift” OR “Chemical Shift Imagings” OR “Imagings, Chemical Shift” OR “Shift Imaging, Chemical” OR “Shift Imagings, Chemical” OR “Chemical Shift Imaging” OR “Tomography, Proton Spin” OR “Proton Spin Tomography” OR “Magnetization Transfer Contrast Imaging” OR “MRI Scans” OR “MRI Scan” OR “Scan, MRI” OR “Scans, MRI” OR “fMRI” OR “MRI, Functional” OR “Functional MRI” OR “Functional MRIs” OR “MRIs, Functional” OR “Functional Magnetic Resonance Imaging” OR “Magnetic Resonance Imaging, Functional” OR “Spin Echo Imaging” OR “Echo Imaging, Spin” OR “Echo Imagings, Spin” OR “Imaging, Spin Echo” OR “Imagings, Spin Echo” OR “Spin Echo Imagings”) AND (“Sensitivity and Specificity” OR Sensitivity OR Specificity).
